# Achieving Enhanced Capacitive Deionization by Interfacial Coupling in PEDOT Reinforced Cobalt Hexacyanoferrate Nanoflake Arrays

**DOI:** 10.1002/gch2.202000128

**Published:** 2021-05-07

**Authors:** Wenhui Shi, Meiting Xue, Xin Qian, Xilian Xu, Xinlong Gao, Dong Zheng, Wenxian Liu, Fangfang Wu, Congjie Gao, Jiangnan Shen, Xiehong Cao

**Affiliations:** ^1^ Center for Membrane and Water Science & Technology College of Chemical Engineering Zhejiang University of Technology 18 Chaowang Road Hangzhou 310014 China; ^2^ College of Materials Science and Engineering Zhejiang University of Technology 18 Chaowang Road Hangzhou 310014 China

**Keywords:** capacitive deionization, faradaic electrodes, metal‐organic frameworks, Prussian blue, water desalination

## Abstract

Capacitive deionization (CDI) as a novel energy and cost‐efficient water treatment technology has attracted increasing attention. The recent development of various faradaic electrode materials has greatly enhanced the performance of CDI as compared with traditional carbon electrodes. Prussian blue (PB) has emerged as a promising CDI electrode material due to its open framework for the rapid intercalation/de‐intercalation of sodium ions. However, the desalination efficiency, and durability of previously reported PB‐based materials are still unsatisfactory. Herein, a self‐template strategy is employed to prepare a Poly(3,4‐ethylenedioxythiophene) (PEDOT) reinforced cobalt hexacyanoferrate nanoflakes anchored on carbon cloth (denoted as CoHCF@PEDOT). With the high conductivity and structural stability achieved by coupling with a thin PEDOT layer, the as‐prepared CoHCF@PEDOT electrode exhibits a high capacity of 126.7 mAh g^−1^ at 125 mA g^−1^. The fabricated hybrid CDI cell delivers a high desalination capacity of 146.2 mg g^−1^ at 100 mA g^−1^, and good cycling stability. This strategy provides an efficient method for the design of high‐performance faradaic electrode materials in CDI applications.

## Graphical abstract

CoHCF@PEDOT nanoflakes anchored on carbon cloth are prepared using a simple self‐template strategy. Combined with the flexibility of the carbon substrate and the high conductivity of Poly(3,4‐ethylenedioxythiophene) (PEDOT), the obtained CoHCF@PEDOT electrode has excellent desalination ability and good cycling stability. This strategy explores a new technique for synthesizing faradaic materials for capacitive deionization applications.

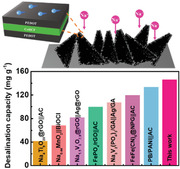

## 1 Introduction

With on‐going population growth and environmental pollution, water scarcity and energy crises have become global issues. The development of water desalination technology plays a vital role in ensuring the sustainable supply of freshwater resources.^[^ ^1^, ^2^, ^3^, ^4^, ^5^, ^6^, ^7^ ^]^ The widely used water desalination technologies mainly include reverse osmosis,^[^ ^8^ ^]^ electrodialysis,^[^ ^9^ ^]^ and multistage flash evaporation.^[^ ^10^ ^]^ However, these technologies generally have problems of high energy consumption, high cost, and secondary pollution.^[^ ^11^ ^]^ Therefore, it is of great importance to develop highly efficient water treatment technology. Capacitive deionization (CDI) is a promising water treatment technology with advantages of low cost, low energy consumption, and easy regeneration of electrodes.^[^ ^12^, ^13^, ^14^, ^15^, ^16^, ^17^ ^]^ In addition to the desalination of brackish water, CDI can also be used in industrial wastewater treatment, water softening, and heavy metal ion removal.^[^ ^18^, ^19^, ^20^, ^21^ ^]^

Carbonaceous materials, such as activated carbon (AC),^[^ ^22^, ^23^ ^]^ carbon aerogel,^[^ ^24^ ^]^ mesoporous carbon,^[^ ^25^ ^]^ carbon nanotubes,^[^ ^26^, ^27^ ^]^ graphene,^[^ ^28^, ^29^ ^]^ and metal‐organic framework (MOF) derived carbon,^[^ ^30^, ^31^ ^]^ are the most typical CDI electrode materials. Since the ions from the feedwater are stored in the electric double layer of the porous structure of carbon electrode, the relatively low charge storage capacity of the carbon material leads to its low desalination capacity.^[^ ^32^ ^]^ In addition, the carbon electrode also faces anodic oxidation reaction, cathodic reduction reaction, and other side reactions such as co‐ion expulsion.^[^ ^33^, ^34^, ^35^ ^]^ Although there are intensive studies on the development of carbon electrode materials and the design of new CDI architecture in recent years, the current CDI technology is still facing problems of low desalination efficiency, and short cycle life.^[^ ^14^, ^36^, ^37^, ^38^ ^]^

CDI systems have many similarities with electrochemical energy storage devices.^[^ ^39^, ^40^, ^41^, ^42^, ^43^, ^44^, ^45^ ^]^ Inspired by battery materials, Lee et al. first applied a typical cathode materials of sodium ion batteries in CDI and proposed a novel hybrid CDI system.^[^ ^46^ ^]^ During desalination process, the Na^+^ ions in the feedwater are captured by the redox reactions in the Na_0.44_MnO_2_ electrode, while the Cl^−^ ions are adsorbed on the surface of the AC electrode. The desalination capacity of the hybrid CDI system surpasses most of the reported carbon electrode materials. In recent years, various faradaic materials have been explored as CDI electrode, which store Na^+^ ions via intercalation or conversion reactions.^[^ ^47^, ^48^, ^49^, ^50^, ^51^, ^52^ ^]^ Owing to the high capacity and high electrochemical stability, these faradaic materials have been considered as promising candidates for next‐generation CDI devices.^[^ ^53^, ^54^ ^]^

Prussian blue (PB) and its analogues (PBA) are a class of MOF materials with formula of A_2_M[Fe(CN)_6_] (A is alkali metal ion, M is transition metal ion).^[^ ^55^, ^56^ ^]^ Different from intercalation type materials, PB has a three‐dimensional (3D) framework with large interstitial voids, which is beneficial to the rapid insertion/extraction of Na^+^ ions.^[^ ^57^, ^58^ ^]^ Among PBA family, hexacyanoferrates (HCFs) are most widely investigated owing to their easy synthesis and low cost.^[^ ^59^, ^60^ ^]^ Recently, PB‐based materials have shown promising performance as CDI electrode. Nevertheless, it still has problems of desalination capacity fading, low coulombic efficiency, and slow desalination rate. Many efforts have been made to improve the sodium storage performance of PB materials.^[^ ^61^, ^62^, ^63^, ^64^ ^]^ One of the typical approaches is to coat or combine with conductive carbon or polymer to enhance the electronic conductivity of PB materials.^[^ ^65^ ^]^ For example, Vafakhah et al. synthesized a highly conductive reduced graphene oxide aerogel embedded with PB nanoparticles as an anode material of CDI.^[^ ^66^ ^]^ The composite aerogel has a large specific surface area and 3D conductive network, which delivered a high desalination capacity of 130 mg g^−1^ at a current density of 100 mA g^−1^. Recently, our group reported a polyaniline tube decorated with PB nanocrystals (PB/PANI composite) by a wet‐chemical approach.^[^ ^67^ ^]^ The obtained PB/PANI composite delivered excellent CDI performance of high desalination capacity (133.3 mg g^−1^ at 100 mA g^−1^), and ultrahigh desalination rate (0.49 mg g^−1^ s^−1^ at 2 A g^−1^), superior to many other recent reports. The enhanced Na capture and desalination mechanism were further revealed via in situ XRD and DFT simulations.

In this work, we design a PEDOT reinforced cobalt HCF (CoHCF) nanoflakes anchored on flexible carbon cloth (denoted as CoHCF@PEDOT) as a free‐standing electrode. The carbon cloth substrate ensures the uniform growth of nanoflakes with no agglomeration. The PEDOT layer is able to enhance the electronic conductivity and reinforce the structural stability of the electrode. As a result, the CoHCF@PEDOT electrode exhibits a high specific capacity of 126.7 mA h g^−1^ at 125 mA g^−1^. By coupling with an AC electrode, the fabricated hybrid CDI cell delivers a high desalination capacity of 146.2 mg g^−1^ at 100 mA g^−1^, and good cycling stability for 50 cycles. This strategy is also applicable to other faradaic electrode materials to boost the desalination performance in CDI.

## 2 Results and Discussion

**Figure 1:**
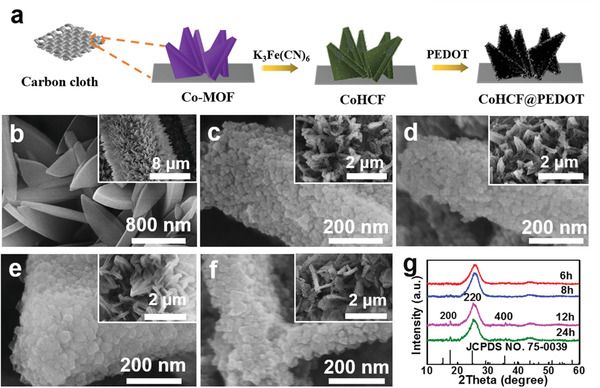
a) Schematic diagram of the preparation procedure of the CoHCF@PEDOT on carbon cloth. b) SEM images of Co‐MOF on carbon cloth. Inset is low‐magnification SEM image. c‐f) SEM images of CoHCF porous nanoflakes obtained at different reaction times: c) 6 h; d) 8 h; e)12 h; and f) 24 h. Insets are corresponding SEM images at low magnification. g) XRD patterns of CoHCF on carbon cloth at different reaction times.

The synthesis process of CoHCF@PEDOT on carbon cloth is illustrated in **Figure** **1**a. First, Co‐MOF nanoflake arrays were uniformly grown on pretreated carbon cloth. Then, Co‐MOF nanoflakes serving as a self‐template were slowly converted into CoHCF nanoflakes. Finally, the PEDOT shell was wrapped on the surface of CoHCF nanoflakes by in situ polymerization of EDOT in chloroform solution. The thin layer of PEDOT was uniformly coated on CoHCF nanoflakes to enhance the conductivity and structural stability.

The microstructure was characterized by scanning electron microscopy (SEM). As shown in Figure 1b, Co‐MOF nanoflakes with a thickness of ≈90 nm were anchored on the carbon cloth fiber. For comparison, Co‐MOF nanoflakes without carbon cloth have much larger size and an average thickness of 460 nm (see Figure S1, Supporting Information). Obviously, the carbon cloth substrate played an important role in not only guiding the in situ growth of Co‐MOF crystals but also effectively decreasing the nanoflake size. After adding [Fe(CN)_6_]^3−^, the Co‐MOF precursors were slowly dissolved and recrystallized to form CoHCF nanocrystals on the surface of nanoflakes. Figure 1c‐f shows the morphology evolution of CoHCF nanoflakes at reaction times from 6 to 24 h. With prolonged reaction time, the shape of parent Co‐MOF precursor was maintained and the obtained CoHCF nanoflakes were composed of nanoparticles.

The crystal structure of as‐prepared Co‐MOF and CoHCF nanoflakes on carbon cloth at different reaction time was confirmed by X‐ray diffraction (XRD). The XRD pattern of Co‐MOF nanoflakes in Figure S2 (Supporting Information) shows the characteristic diffraction peaks of Co‐MOF, which are consistent with previous reports.^[^ ^68^ ^]^ At a reaction time of 6 h, all the characteristic peaks of Co‐MOF disappeared. Upon extended time, the diffraction peaks at 17.5°, 24.9°, and 35.5° were detected, which can be ascribed to the (200), (220), and (400) planes of the CoHCF phase (JCPDS No. 75‐0039).^[^ ^69^ ^]^ The peaks at ≈25° and ≈43° belong to carbon cloth.^[^ ^70^ ^]^ Therefore, the Co‐MOF precursor was completely converted to CoHCF crystals with a reaction time of 12 h.

**Figure 2:**
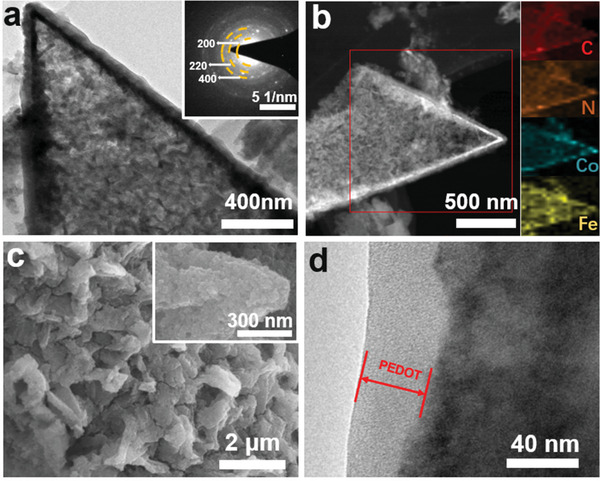
a) TEM image of CoHCF nanoflake. Inset is the corresponding SAED pattern. b) Dark‐field TEM image and corresponding elemental mappings of CoHCF nanoflake. c) SEM image of CoHCF@PEDOT. Inset is high‐magnification SEM image. d) TEM image of CoHCF@PEDOT nanoflake.

The microstructure of the obtained CoHCF at 12 h was further investigated by transmission electron microscope (TEM). CoHCF porous nanoflakes composed with ≈80 nm nanoparticles are presented in **Figure** **2**a, which are well consistent with the SEM observation. The selected area electron diffraction (SAED) pattern confirmed formation of CoHCF phase (inset in Figure 2a). Moreover, energy‐dispersive X‐ray spectroscopy (EDX) elemental mapping in Figure 2b suggests the uniform distribution of C, N, Co, and Fe in the nanoflake. The composition of CoHCF was further examined by X‐ray photoelectron spectroscopy (XPS). Figure S3a (Supporting Information) shows the XPS survey spectrum of CoHCF, suggesting the presence of Co, Fe, C, N, and O elements. Specifically, Figure S3b (Supporting Information) represents the high‐resolution Fe 2p spectrum with two peaks at 721.4 and 708.5 eV, corresponding to Fe 2p_1/2_ and Fe 2p_3/2_ in CoHCF, respectively. Two peaks at 781.6 and 797.2 eV were observed in the high‐resolution Co 2p spectrum, which can be ascribed to Co 2p_1/2_ and Co 2p_3/2_, respectively.^[^ ^71^ ^]^

In addition, a thin layer of PEDOT was uniformly coated on CoHCF nanoflakes to enhance the electrical conductivity and structural stability. The rough surface of CoHCF@PEDOT nanoflakes is observed in SEM image in Figure 2c. TEM image in Figure 2d shows a thin layer of PEDOT with a thickness of ≈40 nm is coated on CoHCF. To further confirm the formation of PEDOT, Fourier transform infrared (FTIR) spectroscopy was conducted on CoHCF and CoHCF@PEDOT. As shown in Figure S4a (Supporting Information), for both CoHCF and CoHCF@PEDOT, the characteristic peaks of CoHCF are observed at 2078 and 591 cm^−1^ corresponding to the bending modes of Co−C≡N−Fe, and the peak at 1570 cm^−1^ belongs to the H−O−H bending mode of H_2_O.^[^ ^72^ ^]^ Moreover, additional peaks of PEDOT appear in CoHCF@PEDOT. The peak at 662 cm^−1^ corresponds to the C−S bond, and 1635 cm^−1^ due to the C−O bond vibration, which is consistent with previous reports.^[^ ^73^, ^74^, ^75^ ^]^ The EDX analysis of CoHCF@PEDOT shows the signals of C, O, S, and Cl elements, suggesting a Cl^−^ doped PEDOT structure (see Figure S4b, Supporting Information). For comparison, bulk CoHCF crystals followed by coating of a PEDOT layer (denoted as bulk‐CoHCF@PEDOT) were also prepared (Figure S5, Supporting Information).^[^ ^73^, ^74^, ^75^ ^]^

**Figure 3:**
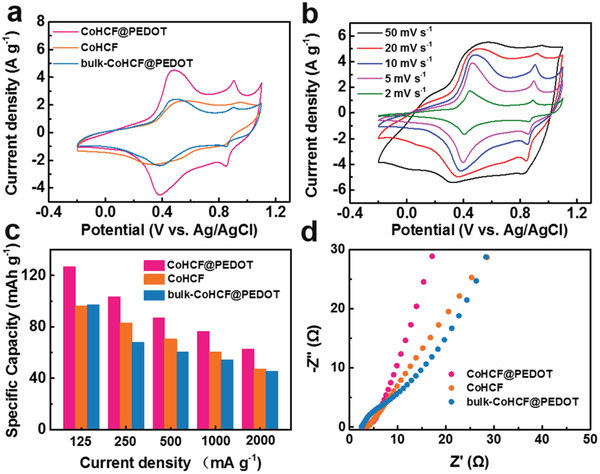
a) CV curves of CoHCF@PEDOT, CoHCF, and bulk‐CoHCF@PEDOT electrodes at a scan rate of 10 mV s^−1^. b) CV curves of CoHCF@PEDOT electrode at different scan rates. c) Specific capacities of CoHCF@PEDOT, CoHCF, and bulk‐CoHCF@PEDOT at different current densities. d) Nyquist plots for CoHCF@PEDOT, CoHCF, and bulk‐CoHCF@PEDOT electrodes.

The electrochemical measurements of CoHCF@PEDOT, CoHCF, and bulk‐CoHCF@PEDOT were performed in a three‐electrode system in 1 mol L^−1^ NaCl solution. As shown in **Figure** **3**a, two pairs of redox peaks at ≈0.3/0.5 and ≈0.85/0.9 V are presented in the cyclic voltammetry (CV) curves, which are related to the reversible insertion and extraction of Na^+^ ions.^[^ ^71^, ^76^ ^]^ Obviously, the CV curve of CoHCF@PEDOT electrode exhibits more pronounced redox peaks and larger integrated area than that of both bulk‐CoHCF@PEDOT and CoHCF, suggesting a higher Na^+^ storage capacity. Meanwhile, the shape of CV curve remained unchanged and peak current increased with increasing the scan rate, indicating an excellent reversibility (Figure 3b). Moreover, the galvanostatic charge/discharge curves of CoHCF, CoHCF@PEDOT, and bulk‐CoHCF@PEDOT electrodes are shown in Figure S6 (Supporting Information). The CoHCF@PEDOT electrode achieved higher capacities than those of CoHCF and bulk‐CoHCF@PEDOT electrodes at current densities ranging from 0.125 to 2 A g^−1^. Notably, the CoHCF@PEDOT electrode exhibits a high specific capacity of 126.7 mAh g^−1^ at 125 mA g^−1^ (Figure 3c). The superior performance of CoHCF@PEDOT electrode was further investigated by electrochemical impedance spectroscopy (EIS) measurements. Figure 3d shows the Nyquist plots for CoHCF, CoHCF@PEDOT, and bulk‐CoHCF@PEDOT electrodes, which suggest that the CoHCF@PEDOT electrode displays lower charge transfer resistance and faster ion diffusion rate than CoHCF and bulk‐CoHCF@PEDOT electrodes.

**Figure 4:**
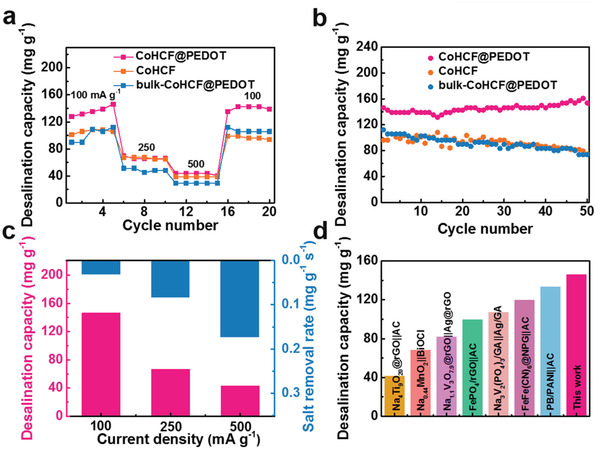
a) Desalination capacities of CoHCF@PEDOT, CoHCF, and bulk‐CoHCF@PEDOT electrodes at various current densities. b) Cycling performance of CoHCF@PEDOT, CoHCF, and bulk‐CoHCF@PEDOT electrodes at a current density of 100 mA g^−1^. c) The desalination capacity and corresponding salt removal rate of CoHCF@PEDOT at various current densities. d) Comparison of desalination performances of recently reported Faradaic electrode materials.

The potential application of CoHCF@PEDOT in CDI was further explored due to its excellent electrochemical performance. As shown in Figure S7 (Supporting Information), the CDI cell was assembled with CoHCF@PEDOT electrode as cathode and AC electrode as anode. During the CDI desalination process, Na^+^ ions are intercalated into the CoHCF@PEDOT electrode, whereas Cl^−^ ions are captured by the AC electrode, respectively. **Figure** **4**a compares the CDI desalination capacities of the CoHCF@PEDOT, CoHCF, and bulk‐CoHCF@PEDOT electrodes at various current densities in 750 mg L^−1^ NaCl solution. It can be seen that CoHCF@PEDOT electrode achieved a desalination capacity of 146.2 mg g^−1^ at 100 mA g^−1^, which is much higher than those of CoHCF electrode (108.2 mg g^−1^) and bulk‐CoHCF@PEDOT electrode (112.2 mg g^−1^). The CoHCF@PEDOT electrode can still achieve a desalination capacity of 43.13 mg g^−1^ at a high current density of 500 mA g^−1^. In addition, the CoHCF@PEDOT electrode shows an excellent stability with ≈100% retention of desalination capacity for 50 cycles at a current density of 100 mA g^−1^ (Figure 4b). However, significant decay of desalination capacities is observed for both CoHCF and bulk‐CoHCF@PEDOT electrodes. The stable cycling performance is ascribed to the structural stability of the CoHCF@PEDOT electrode. As shown in Figure S8 (Supporting Information), the corresponding charge efficiency of the CoHCF@PEDOT electrode remains stable at ≈90% over 50 cycles, which is higher than that of CoHCF (≈80% after 50 cycles) and bulk‐CoHCF@PEDOT (≈80% after 50 cycles). In addition to desalination capacity, salt removal rate is another important parameter for the electrode materials applied in CDI. Figure 4c shows the desalination capacity and salt removal rate of the CoHCF@PEDOT electrode. A high salt removal rate of 0.031 mg g^−1^ s^−1^ is exhibited for the CoHCF@PEDOT electrode at a current density of 100 mA g^−1^. As shown in Figure 4d and Table S1 (Supporting Information), the desalination performance of CoHCF@PEDOT electrode is among the best of recently reported faradaic electrodes for CDI.^[^ ^67^, ^77^, ^78^, ^79^, ^80^, ^81^, ^82^, ^83^, ^84^, ^85^, ^86^ ^]^

## 3 Conclusion

In summary, a hierarchical structure of CoHCF@PEDOT nanoflakes anchored on carbon cloth was synthesized. Through forming a stable porous structure coated with a conductive polymer, the CoHCF@PEDOT exhibits an outstanding specific capacity (126.7 mA h g^−1^ at 125 mA g^−1^) in the 1 mol L^−1^ NaCl electrolyte. When used as the CDI electrode, CoHCF@PEDOT obtains an excellent desalination capacity of 146.2 mg g^−1^ at 100 mA g^−1^, and good cycling stability for 50 cycles. This strategy is good for the development of high‐performance faradaic electrode materials in CDI applications.

## 4 Experimental Section

### Synthesis of Co‐MOF

The carbon cloth was first treated in a mixture of nitric acid and sulfuric acid at 80 °C for 4 h, followed by thorough washing and drying. The pretreated carbon cloth with a size of ≈3 × 3 cm^2^ was immersed in DI water (40 mL) containing cobalt nitrate (0.589 g) with stirring. Then, 2‐methylimidazole (1.3 g) was dissolved in DI water (40 mL) and quickly poured into above solution and stirred for 2 h at room temperature. The carbon cloth with Co‐MOF was rinsed with DI water and ethanol, followed by drying at 70 °C for 12 h in a vacuum oven. For comparison, Co‐MOF nanoflakes were synthesized by the same method without using carbon cloth.

### Synthesis of CoHCF

The as‐prepared Co‐MOF on carbon cloth was immersed in ethanol (20 mL). Then, aqueous solution of potassium ferricyanide (K_3_Fe(CN)_6_) (10 mg, 20 mL) and HCl solution (1 mol L^−1^, 50 µL) was slowly added into the above ethanol solution under stirring, and kept at 25 °C for 6 to 24 h. The obtained product was washed with DI water and ethanol, and then dried at 70 °C for 12 h to get CoHCF nanoflakes on carbon cloth. For comparison, bulk‐CoHCF was also synthesized. Typically, cobalt chloride hexahydrate (0.143 g) and sodium citrate dihydrate (0.397 g) were dissolved in DI water (20 mL). Then, the above solution was slowly added into aqueous solution containing potassium ferricyanide (0.132 g, 20 mL). The mixture was stirred at room temperature for 6 h. The obtained product was washed with water and ethanol, and then dried in a vacuum oven at 70 °C for 12 h.

### Synthesis of CoHCF@PEDOT

EDOT (5 mg) was first dissolved in chloroform (20 mL) in an ice bath. Then FeCl_3_ (20 mg) and obtained CoHCF on carbon cloth were added into the above solution. After stirring for 6 h, the product was rinsed with water repeatedly, and dried at 60 °C overnight in a vacuum oven to get CoHCF@PEDOT on carbon cloth. Bulk‐CoHCF@PEDOT was also synthesized by the same method without carbon cloth.

### Characterization

FE‐SEM (Hitachi SU‐8010) and TEM (JEM‐100CX II) equipped with EDX were employed to characterize the morphologies of the obtained samples. The composition of samples was analyzed by X‐ray diffractometer (PANalytical X'Pert Pro, Cu Kα‐irradiation, λ = 0.15 404 nm) and XPS (Thermo Scientific, ESCALAB250Xi). The FTIR spectroscopy was conducted on a FTIR spectrometer (Nicolet, iS50).

### Electrochemical Measurements

The electrochemical performance of electrode was measured in a three‐electrode system with Pt as the counter electrode and Ag/AgCl as reference electrode and NaCl solution (1 mol L^−1^) as electrolyte. An electrochemical workstation (Metrohm, Autolab PG302N) was employed to conduct CV, galvanostatic charge/discharge and EIS. The CoHCF@PEDOT on carbon cloth (≈2 mg of mass loading on 1 × 1 cm^2^) was directly used as working electrode. The working electrode of bulk‐CoHCF@PEDOT was prepared by mixing with polyvinylidene fluoride and carbon black at a mass ratio of 8:1:1 in 1‐methyl‐2‐pyrrolidinone (NMP) solution and coated on carbon cloth, followed by drying overnight at 60 °C.

### Measurements of CDI Performance

The desalination test was performed in a hybrid CDI cell, which was composed of an active material electrode, an AC electrode, a cation exchange membrane (CEM, ASTOM Corp, Japan), and an anion exchange membrane (AEM, ASTOM Corp, Japan). The electrode was composed of 3 × 3 cm^2^ carbon cloth with ≈10 mg of mass loading of active materials. Acrylic sheets with flowing channels in the center were used as spacer. Constant current was applied on the CDI cell during the desalination and regeneration process. NaCl solution (750 mg L^−1^) was prepared as feed water and the flow rate was kept at 50 mL min^−1^. The conductivity of the NaCl solution was recorded by a conductivity meter (Leici, DDSJ‐308F). The desalination capacity and salt removal rate were calculated by the following equations:

(1) $$\begin{array}{c} \Gamma =\frac{\left(C_{o}-C_{e}\right)V}{m} \end{array}$$

(2) $$\begin{array}{c} \nu =\frac{\Gamma }{t} \end{array}$$

where Γ (mg g^−1^) is the desalination capacity, *C_o_* (mg L^−1^) and *C_e_* (mg L^−1^) are the initial and final concentrations of NaCl solution, *V* (L) is the volume of feed water solution, *m* (g) is the total mass of electrodes, v (mg g^−1^ s^−1^) is the salt removal rate, *t* (s) is the desalination time.

## Conflict of Interest

The authors declare no conflict of interest.

## Supporting information

Supporting Information

## Data Availability

Research data are not shared.
