# Supplementary material for: Achieving Enhanced Capacitive Deionization by Interfacial Coupling in PEDOT Reinforced Cobalt Hexacyanoferrate Nanoflake Arrays
Source: Glob Chall. 2021 May 7;5(8):2000128. doi: 10.1002/gch2.202000128 (PMC8335821; doi:10.1002/gch2.202000128)
Supplement: Supplementary file 1 — Supporting Information [file GCH2-5-2000128-s001.pdf]

© 2021 Wiley-VCH GmbH

Global Challenges
Open Access

Supporting Information

for *Global Challenges,* DOI: 10.1002/gch2.202000128

Achieving Enhanced Capacitive Deionization by Interfacial Coupling in PEDOT Reinforced Cobalt Hexacyanoferrate Nanoflake Arrays

Wenhui Shi, Meiting Xue, Xin Qian, Xilian Xu, Xinlong Gao, Dong Zheng, Wenxian Liu, Fangfang Wu, Congjie Gao, Jiangnan Shen,* and Xiehong Cao**

© 2021 Wiley-VCH GmbH

Supporting Information

Achieving Enhanced Capacitive Deionization by Interfacial Coupling in PEDOT Reinforced Cobalt Hexacyanoferrate Nanoflake Arrays

Wenhui Shi, Meiting Xue, Xin Qian, Xilian Xu, Xinlong Gao, Dong Zheng, Wenxian Liu, Fangfang Wu, Congjie Gao, Jiangnan Shen,* and Xiehong Cao**

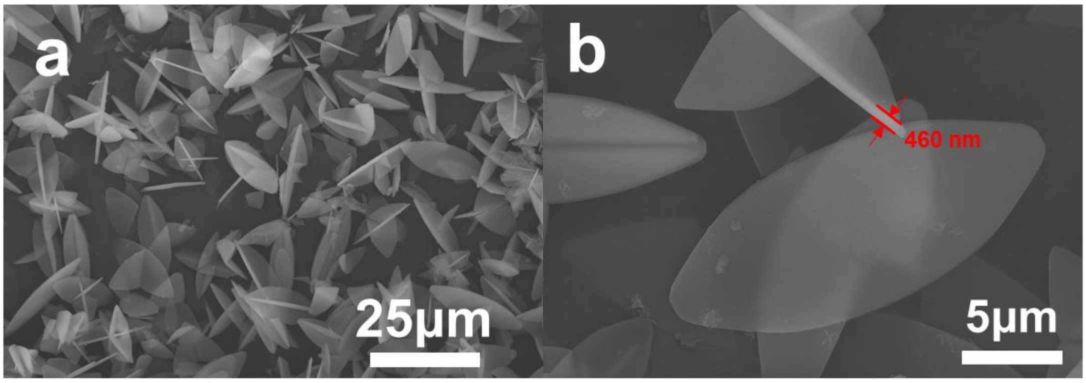

Figure S1. (a, b) SEM images of Co-MOF nanoflakes without carbon cloth.

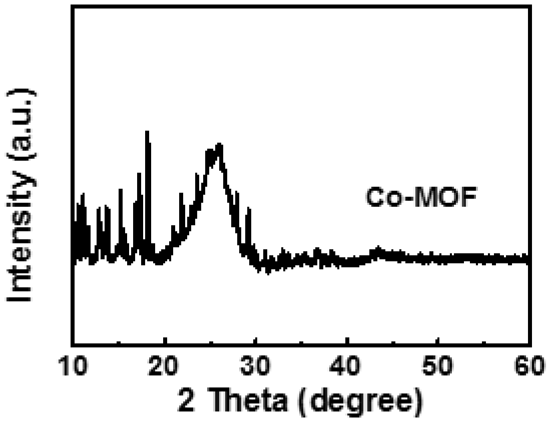

Figure S2. XRD pattern of Co-MOF on carbon cloth.

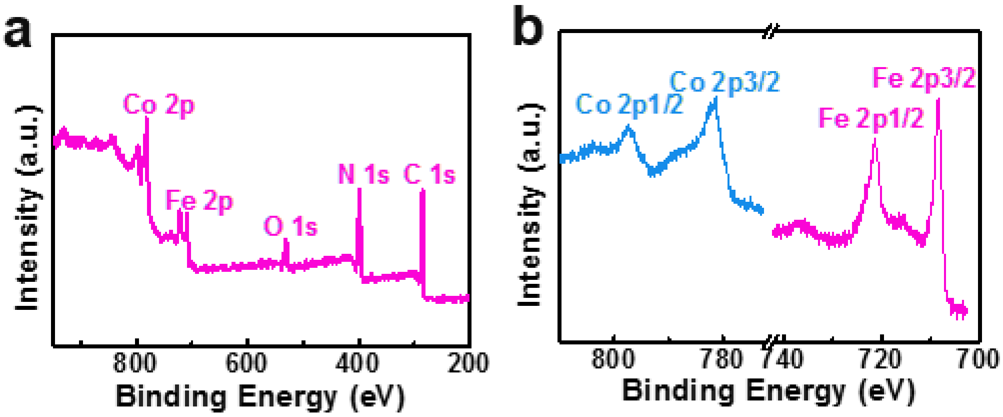

Figure S3. (a) XPS survey spectrum and (b) high-resolution spectra of Co 2p and Fe 2p for CoHCF nanoflakes.

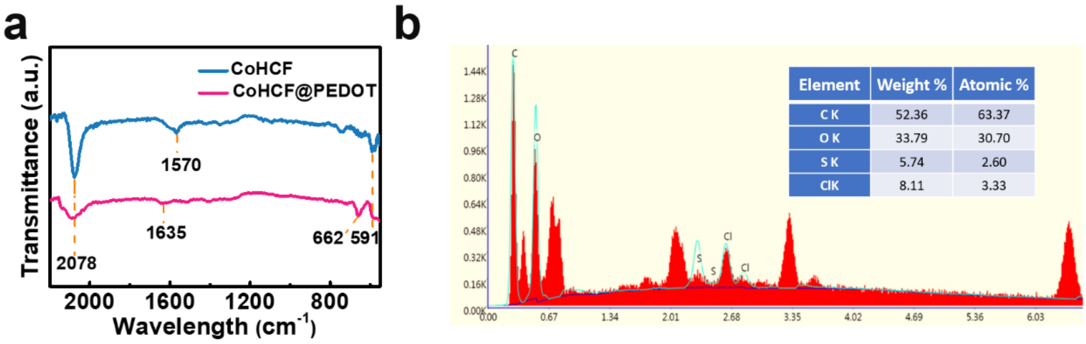

Figure S4. (a) FTIR spectra of CoHCF andCoHCF@PEDOT. (b) The EDX spectrum of CoHCF@PEDOT.

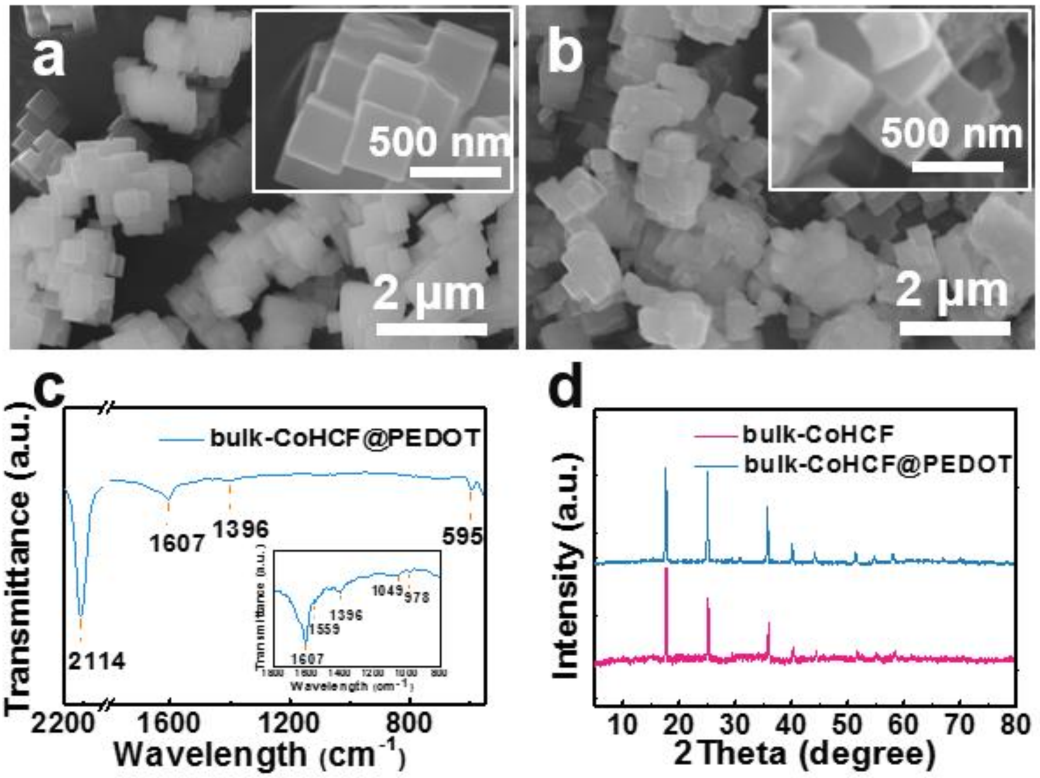

Figure S5. (a, b) SEM images of bulk-CoHCF and bulk-CoHCF@PEDOT at different magnifications. (c) FTIR spectra of bulk-CoHCF@PEDOT. (d) XRD patterns of bulk-CoHCF and bulk-CoHCF@PEDOT.

From the FTIR spectra of bulk-CoHCF@PEDOT, we can know that the absorption peaks of bulk-CoHCF@PEDOT at 978, 1049 1396, 1559 and 1607 cm^{-1} characterizes the C–S, C-O-C,C–C, C=C and C-O bond vibration corresponding to the PEDOT, the peaks in 2114 and 595 cm^{-1} belong to the bending modes of Co−C≡N−Fe from bulk-CoHCF.

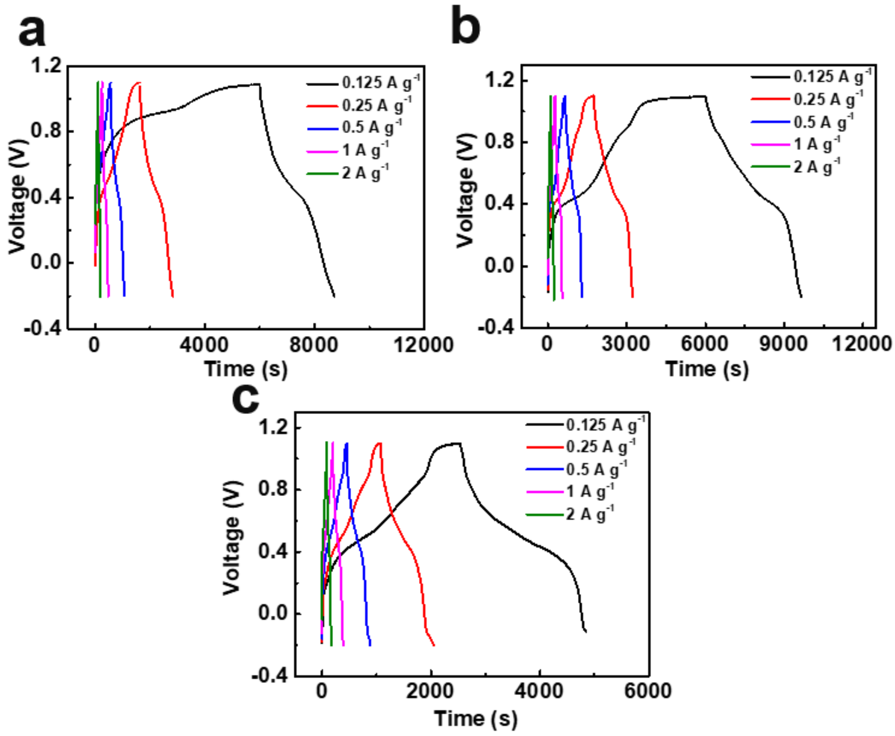

Figure S6. Charge-discharge plots of (a) CoHCF, (b) CoHCF@PEDOT and (c) bulk-CoHCF@PEDOT at different current density.

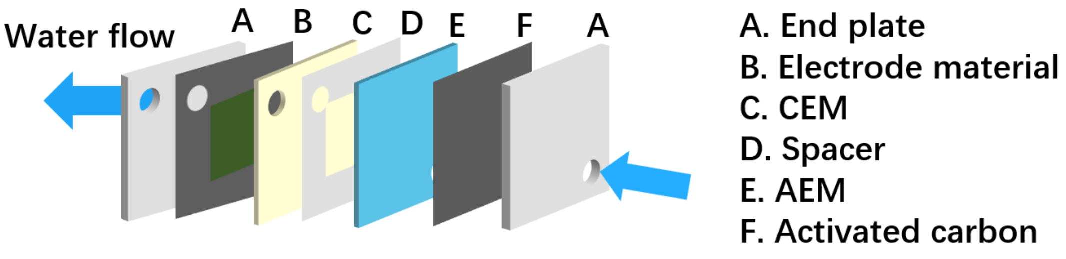

Figure S7. A schematic diagram of the CDI cell.

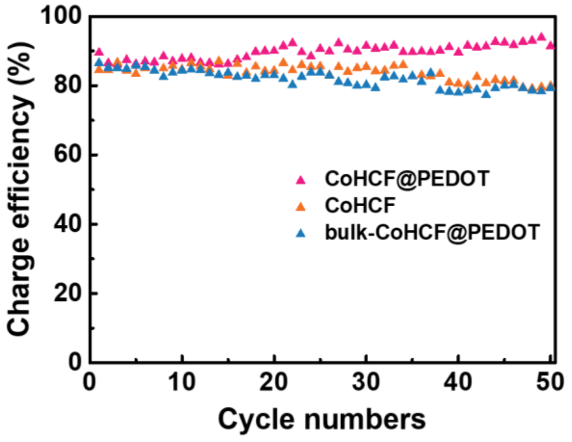

Figure S8. The corresponding charge efficiency of CoHCF@PEDOT, CoHCF and bulk-CoHCF@PEDOT electrodes at a current density of 100 mA g^{-1}.

Table S1. Comparison of the desalination performance of the CoHCF@PEDOT electrode with recently reported faradaic electrode materials.

Electrode materials	Initial Salt Concentration (mg L^{-1})	Desalination Capacity (mg g^{-1})	Desalination Rate (mg g^{-1} s^{-1})	Current density/ Voltage	Ref
PB/PANI ||AC	1000	133.9	0.370	500mA g^{-1}	*Adv. Mater.* **2020,** *32* (33), 1907404.
$Na_3V_2(PO_4)_3$/ GA||Ag/GA	1000	107.5	0.0940	100mA g^{-1}	*Small* **2019,** *15* (9), 1805505.
$Na_3V_2(PO_4)_3$ @C||AC	585	34	0.0400	1.0 V	Nano Lett. **2019**, 19, 823.
α-MnO_2 ||AC	877	29.1	0.140	1.2 V	*Nano Energy* **2018,** *44*, 476-488.
$Na_{0.44}MnO_2$|| BiOCl	760	68.5	0.092	100mA g^{-1}	*Energy Environ. Sci.* **2017,** *10* (10), 2081-2089.
$Na_4Ti_9O_{20}$@ rGO||AC	250	41.8	0.0116	1.4 V	*Chem. Eng. J.* **2018,** *343,* 8-15.
MoS_2/g-C_3N_4 ||MoS_2 /g-C_3N_4	250	24.16	0.00671	1.6 V	*Desalination* **2020,** *479*, 114348.
$FePO_4$/rGO ||AC	700	100	0.117	100mA g^{-1}	*J. Mater. Chem. A* **2018,** *6* (19), 8901-8908.
$Ti_3C_2T_x$ ||$Ti_3C_2T_x$	300	14	0.0160	1.2 V	*J. Mater. Chem. A* **2016,** *4* (47), 18265-18271
$FeFe(CN)_6$@ NPG||AC	765	120	0.293	125mA g^{-1}	*Nanoscale* **2017,** *9* (35), 13305-13312.
$Na_{1.1}V_3O_{7.9}$ @rGO|| Ag@rGO	2000	82.2	0.0228	1.4 V	*J. Mater. Chem. A* **2019,** *7* (28), 16892-16901.
**CoHCF@ PEDOT||AC**	**750**	**146.2**	**0.172**	**100mA g^{-1}**	**This Work**
